# Impaired Levels of Gangliosides in the Corpus Callosum of Huntington Disease Animal Models

**DOI:** 10.3389/fnins.2016.00457

**Published:** 2016-10-06

**Authors:** Alba Di Pardo, Enrico Amico, Vittorio Maglione

**Affiliations:** Istituto Neurologico Mediterraneo (IRCCS) NeuromedPozzilli, Italy

**Keywords:** HD, Gangliosides, GM1, GD1a, GT1b, Corpus Callosum White Matter (CC-WM)

## Abstract

Huntington Disease (HD) is a genetic neurodegenerative disorder characterized by broad types of cellular and molecular dysfunctions that may affect both neuronal and non-neuronal cell populations. Among all the molecular mechanisms underlying the complex pathogenesis of the disease, alteration of sphingolipids has been identified as one of the most important determinants in the last years. In the present study, besides the purpose of further confirming the evidence of perturbed metabolism of gangliosides GM1, GD1a, and GT1b the most abundant cerebral glycosphingolipids, in the striatal and cortical tissues of HD transgenic mice, we aimed to test the hypothesis that abnormal levels of these lipids may be found also in the corpus callosum white matter, a ganglioside-enriched brain region described being dysfunctional early in the disease. Semi-quantitative analysis of GM1, GD1a, and GT1b content indicated that ganglioside metabolism is a common feature in two different HD animal models (YAC128 and R6/2 mice) and importantly, demonstrated that levels of these gangliosides were significantly reduced in the corpus callosum white matter of both models starting from the early stages of the disease. Besides corroborating the evidence of aberrant ganglioside metabolism in HD, here, we found out for the first time, that ganglioside dysfunction is an early event in HD models and it may potentially represent a critical molecular change influencing the pathogenesis of the disease.

## Introduction

Alterations in lipid metabolism have been recently recognized as a novel molecular hallmark that may profoundly affect brain homeostasis in Huntington's disease (HD), a rare genetic disorder characterized by the progressive neurodegeneration and associated motor, cognitive and behavioral disturbances (The Huntington's Disease Collaborative Research Group, 1993; Novak and Tabrizi, 2011). The disease-causing mutation is a CAG repeat expansion within the gene encoding huntingtin (Htt) protein, whose mutated form exerts a variety of undesirable toxic effects in both neuronal and non-neuronal cells (Bradford et al., 2009, 2010; Hsiao and Chern, 2010). Several recent studies have indeed largely described oligodendrocytes defects in HD and highlighted a correlation with cerebral white matter (WM) disorganization either in animal models or in human patients with the disease (Fennema-Notestine et al., 2004; Ciarmiello et al., 2006; Xiang et al., 2011; Di Paola et al., 2012, 2014; Huang et al., 2015; Jin et al., 2015; Southwell et al., 2015; Gatto et al., 2015). The nature of such defects might hypothetically be attributable to an altered lipid/ganglioside composition of the same brain structures, however much remains to be elucidated.

Ganglioside GM1, GD1a, and GT1b the most abundant glycosphingolipids in the Central Nervous System (CNS), are normally implicated in several physiological events including regulation of either neuronal or non-neuronal cell function, maintenance of myelinated fibers and white matter integrity (Kim, 1990; Posse de Chaves and Sipione, 2010; Schnaar, 2010). Defective ganglioside content has been widely associated with white matter abnormalities. Mice lacking some of the enzymes controlling the synthesis of all three gangliosides showed axonal degeneration, CNS white matter vacuolization, perturbed myelin paranodal stability and disruption of axonal-glia interaction (Sheikh et al., 1999; Yamashita et al., 2005; Sabourdy et al., 2008).

Many previous studies have reported disturbance in ganglioside metabolism in HD in different brain regions in both animal models and patients with the disease (Desplats et al., 2007; Denny et al., 2010; Maglione et al., 2010; Di Pardo et al., 2014). However, these past studies did not explore the potential deregulation of this metabolism in the corpus callosum white matter (CC-WM), where these lipids are recognized to play a critical role.

White matter abnormalities have been extensively reported in HD even before overt striatal neuronal loss or occurrence of clinical signs in both animal models and human patients (Ciarmiello et al., 2006; Lerch et al., 2008; Di Paola et al., 2012, 2014; Poudel et al., 2014) however, no definitive hypothesis conceivably explaining the type of dysfunction or its nature has been advanced so far.

In this study, besides further consolidating the evidence of aberrant ganglioside metabolism in the striatal and cortical tissues of HD mice, we extended the analysis also to the cerebral CC-WM with the aim of finding out any possible link between lipid compositional perturbations and white matter alterations (Fennema-Notestine et al., 2004; Ciarmiello et al., 2006; Xiang et al., 2011; Di Paola et al., 2012, 2014; Huang et al., 2015; Jin et al., 2015; Southwell et al., 2015; Gatto et al., 2015). Interestingly, in line with our hypothesis our findings demonstrated for the first time that ganglioside content is abnormal in the CC-WM of HD models and its deregulation occurs early in the disease.

## Materials and methods

### Animal models

Both R6/2 (carrying approximately 160 ± 5 CAG repeat expansion) and YAC128 HD mouse colonies were housed in the animal facility at IRCCS Neuromed. All animal studies were performed in accordance with approved protocols by the IRCCS Neuromed Animal Care Review Board and by “Istituto Superiore di Sanità” (permit number: 1163/2015- PR) and were conducted according to EU Directive 2010/63/EU for animal experiments. All the analyses were carried out in pre-symptomatic (4 week old R6/2; 2.5 month old YAC128), early symptomatic (6 week old R6/2; 5 month old YAC128) and symptomatic HD mice (12 week old R6/2; 9 month old YAC128) as well as in age-matched wild-type (WT) littermates.

### Total lysate preparation

Mice were first sacrificed by cervical dislocation and brains were removed from the skull. Brains were split in two hemispheres and ventral sides were placed up, pons was then lifted and hippocampus removed. Subsequently, CC-WM was carefully and gently lifted away from the underlying cortex and collected from both hemispheres (Supplementary Figure 1). Cortex and striatum were finally isolated and placed in separate eppendorf tubes. All brain regions were snap frozen in liquid N2 and pulverized in a mortar with a pestle. Tissues were homogenized in lyses buffer containing 20 mM Tris, pH 7.4, 1% Nonidet P-40, 1 mM EDTA, 20 mom NaF, 2 mM Na3V04, and 1:1000 protease inhibitor mixture (Sigma-Aldrich) and sonicated with 2 × 10 s pulses. Tissue lysates were clarified by centrifugation at 10,000 × g for 15 min at 4°C. Protein concentration was determined by Bradford method (Bio-Rad Laboratories).

### Analysis of ganglioside content in mouse brains

To assure that equal amount of homogenate was analyzed, each sample tissue lysate was serially diluted and protein concentration was re-assessed by NanoDrop Spectrophotometer. Fifty picograms of total protein lysates, from both R6/2 and YAC128 HD and control mice were then spotted in quadruplicates on nitrocellulose membrane and, dot-blotting analysis was performed as previously reported (Di Pardo et al., 2014). For GM1 quantitation, membranes were then blocked in 5% milk in TBS-T and incubated with HRP-conjugated cholera toxin subunit B (5 μg/mL) (Invitrogen C34780; lot number: 1306570) for 30 min at room temperature. For GD1a and GT1b gangliosides, membranes were incubated for 3 h at room temperature with anti-GD1a (1:5000) (Millipore MAB5606; lot number: 2199592) and anti-GT1b (1:5000) (Millipore MAB5608; lot number: 2361832), respectively. For GD1a and GT1b a goat anti-mouse Gig HRP-conjugated secondary antibody (1:5000) (Santa Cruz sc-2005; lot number: B0813) was used. Ganglioside spots were detected by ECL Prime (GE Healthcare) and quantitated with Quantity One (Bio-Rad Laboratories).

### Statistics

Non-parametric Mann Whitey U was used to analyze ganglioside content in all experiments. All data were expressed as mean ± SD.

## Results

### Ganglioside levels are perturbed in the CC-WM from symptomatic YAC128 mice

**Figure 1:**
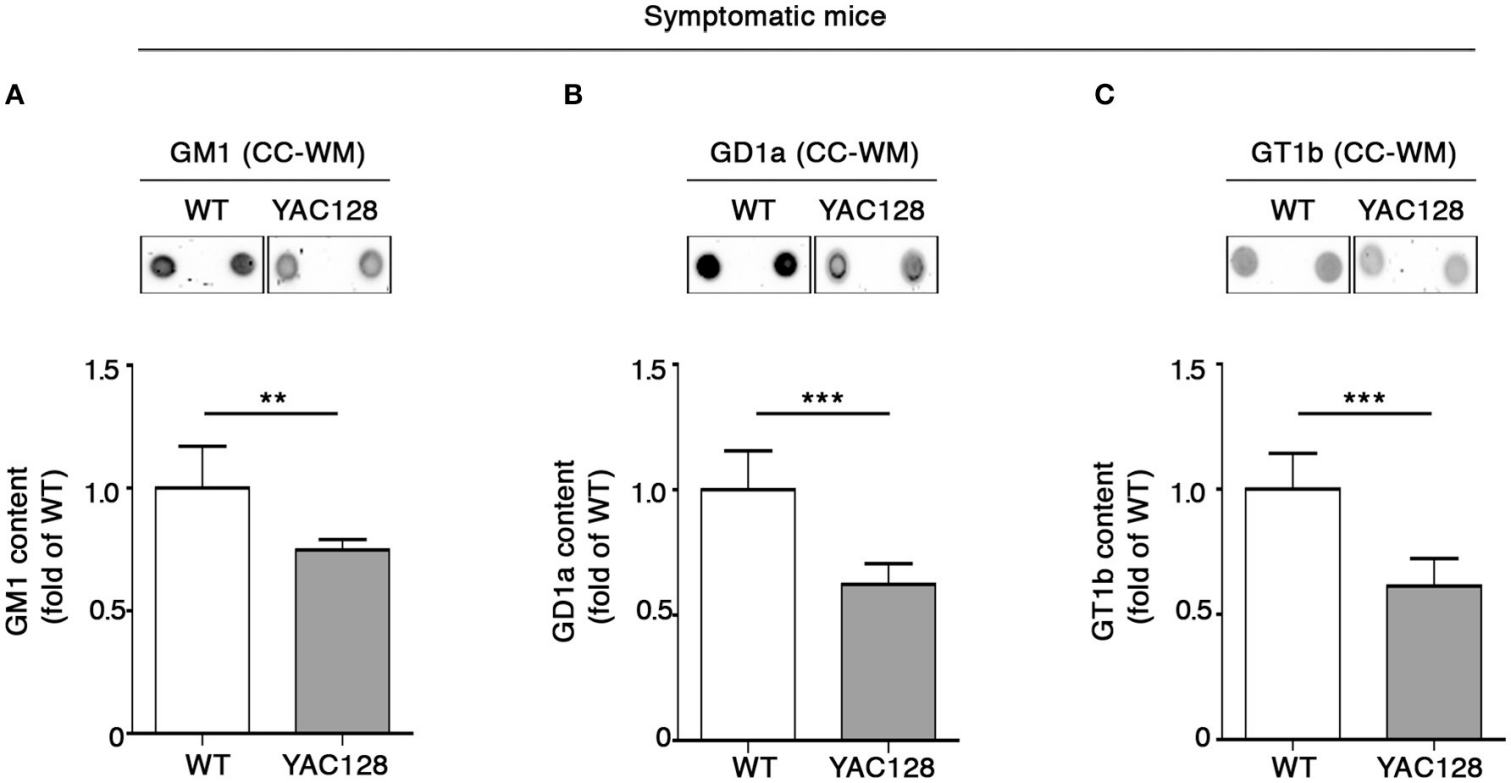
**Brain ganglioside content is reduced in CC-WM of symptomatic YAC128 HD mice**. Representative dot blotting and densitometric analysis of gangliosides GM1 **(A)**, GD1a **(B)**, and GT1b **(C)** in CC-WM from symptomatic YAC128 (9 month old) mice and age-matched WT littermates. Ganglioside spots were visualized by ECL. Data are represented as the mean ± SD, *n* = 5 for each group of mice. ^**^P < 0.001; ^***^P < 0.0001 (non-parametric Mann–Whitney *U*-test).

Perturbed ganglioside metabolism in the YAC128 mouse model was first described by using the Thin Layer Chromatography (TLC) in the striatal and cortical tissues from symptomatic mice (Maglione et al., 2010), however, no attention was paid to ganglioside content in the CC-WM. In order to provide a more complete scenario about ganglioside profile in HD mice, here, besides having further confirmed, with a different technical approach (dot blotting), the alteration of GM1, GD1a, and GT1b levels in both striatum (Supplementary Figures 2A–C), and cortex (Supplementary Figures 2D–F) of YAC128 mice, we examined ganglioside content also in the CC-WM of the same animals. Interestingly, dot-blotting analysis showed that all three gangliosides were significantly reduced in this specific brain region in symptomatic YAC128 mice when compared to age-matched WT littermates (Figures 1A–C).

### Alteration of ganglioside metabolism is not confined to perturbed GM1 content in the striatum of symptomatic R6/2 mice

**Figure 2:**
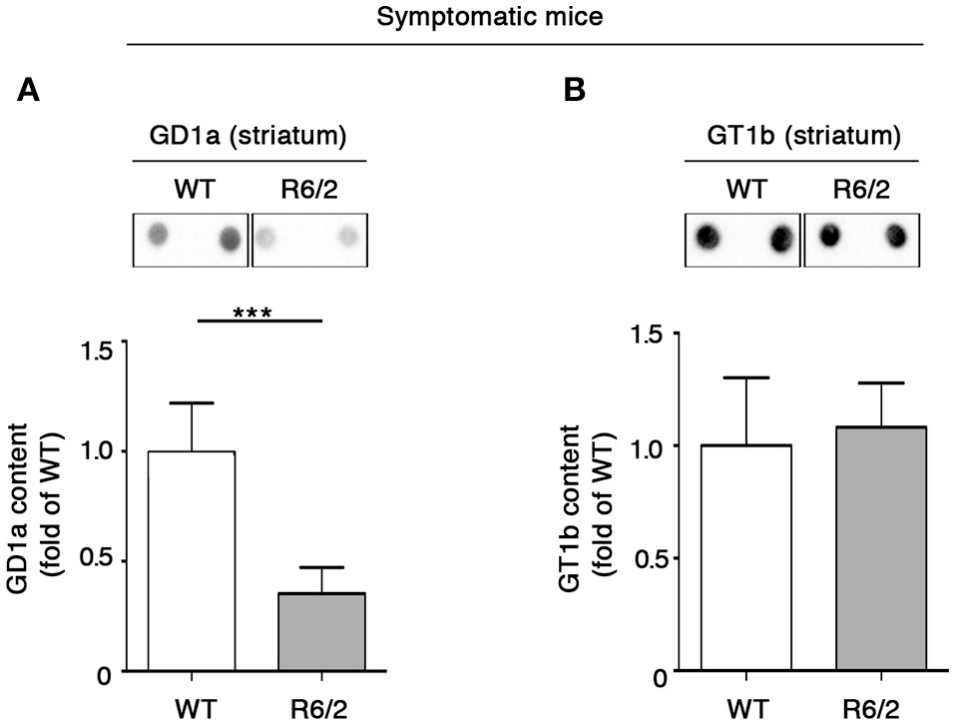
**Levels of ganglioside GD1a are reduced in the striatum of symptomatic R6/2 mice**. Representative dot blotting and densitometric analysis of GD1a **(A)** and GT1b **(B)** in striatal tissues isolated from symptomatic R6/2 mice and WT controls. Ganglioside spots were visualized by ECL. Data are represented as the mean ± SD, *n* = 7 for each group of mice. ^***^P < 0.0001 (non-parametric Mann–Whitney *U*-test).

The finding of GM1 reduction in the striatum of symptomatic R6/2 mice (Di Pardo et al., 2014 and Supplementary Figure 3) corroborated the idea of dysfunctional ganglioside metabolism in HD (Maglione et al., 2010), however did not clarify to what extend other gangliosides might be also affected in this HD animal model. Thus, with the aim of addressing this issue, ganglioside GD1a and GT1b levels were measured in the same brain tissue of the same mice. Semi-quantitative analysis of ganglioside content indicated a marked decrease in GD1a levels in symptomatic (12 week old) R6/2 mice compared to age-matched control mice (Figure 2A), whereas no changes were detected in GT1b content (Figure 2B).

**Figure 3:**
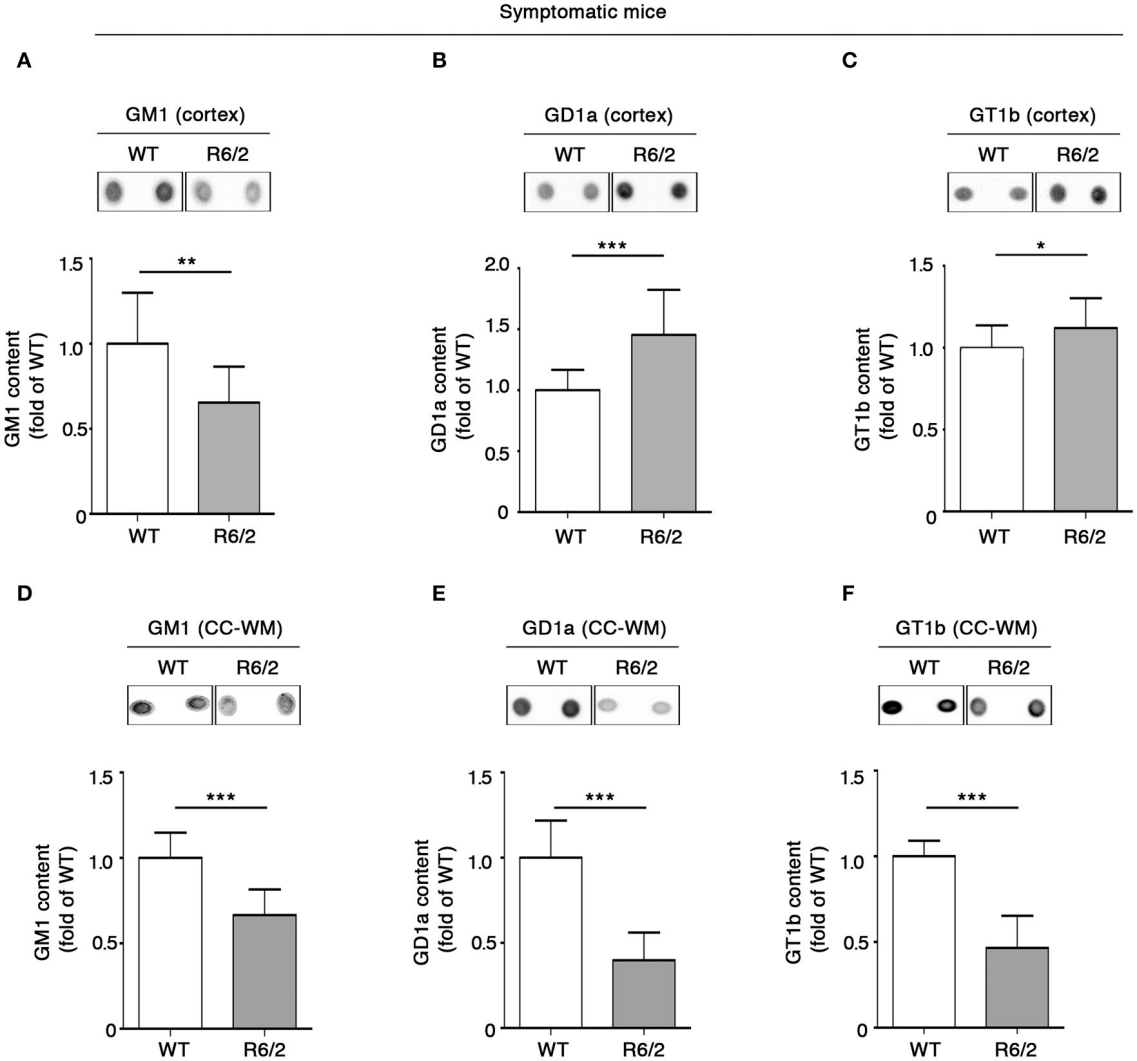
**Levels of brain gangliosides are aberrant also in the cortex and CC-WM from symptomatic R6/2 HD mice**. Representative dot blotting and densitometric analysis of gangliosides GM1, GD1a, and GT1b in cortex **(A–C)** and CC-WM **(D–F)** from symptomatic (12 week old) R6/2 mice and age-matched WT littermates. Ganglioside spots were visualized by ECL. Data are represented as the mean ± SD, *n* = 7 for each group of mice. ^*^P < 0.05; ^**^P < 0.001; ^***^P < 0.0001 (non-parametric Mann–Whitney *U*-test).

Also, we extended the analysis of ganglioside GM1, GD1a, and GT1b content to different other brain areas of the same mice. Consistent with reduced levels in the striatal tissues, GM1 was considerably decreased also in the cortical tissues (Figure 3A). However, a different profile was observed for GD1a and GT1b, whose levels were significantly increased in R6/2 compared to WT control littermates (Figures 3B,C).

Interestingly, when ganglioside analysis was applied to CC-WM of the same symptomatic R6/2 mice, a dramatic reduction in the levels of all three gangliosides was detected (Figures 3D–F).

### Early ganglioside perturbation in both R6/2 and YAC128 mice

**Figure 4:**
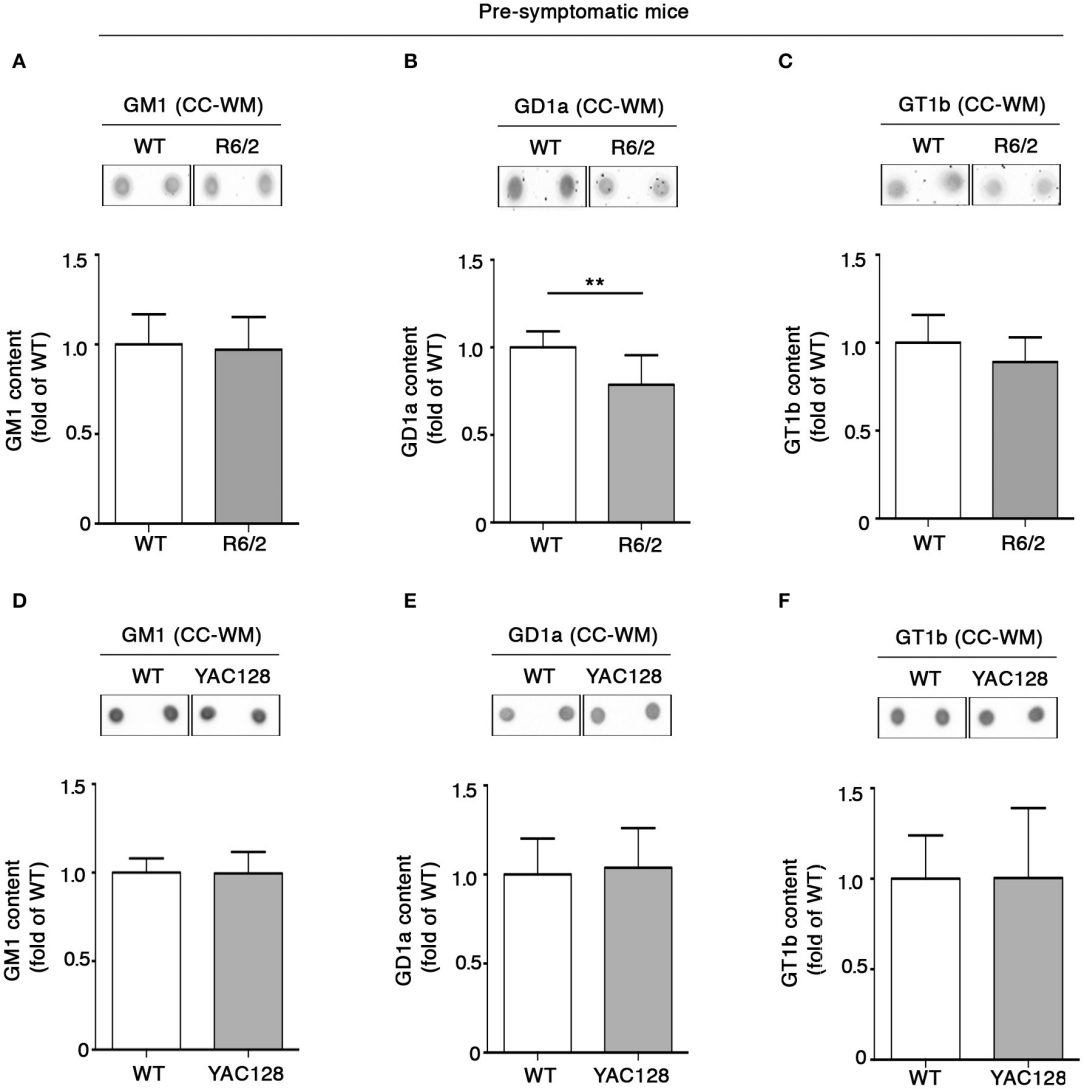
**Levels of ganglioside GD1a are reduced in CC-WM levels from pre-symptomatic R6/2 HD mice**. Representative dot blotting and densitometric analysis of gangliosides GM1, GD1a, and GT1b in CC-WM from pre-symptomatic R6/2 (4 week old) **(A–C)** and YAC128 (2.5 month old) **(D–F)** and age-matched WT littermates. Ganglioside spots were visualized by ECL. Data are represented as the mean ± SD, *n* = 5 for each group of mice. ^**^P < 0.001 (non-parametric Mann–Whitney *U*-test).

In order to investigate whether the early white matter abnormalities previously described in HD models (Lerch et al., 2008; Xiang et al., 2011; Gatto et al., 2015; Jin et al., 2015), may be associated with any potential alteration of lipid composition, levels of gangliosides GM1, GD1a, and GT1b in the CC-WM of both pre-symptomatic and early symptomatic R6/2 and YAC128 mice were determined. Interestingly, warning signs of dysfunctional ganglioside metabolism were detected at the pre-symptomatic stage before any visible disease symptoms in R6/2 mice. While the concentration of both GM1 and GT1b did not change between pre-symptomatic R6/2 mice (4 week old) and age-matched controls (Figures 4A–C), GD1a content was significantly reduced in R6/2 animals when compared to WT littermates (Figure 4B). A different scenario emerged in the CC-WM from pre-symptomatic YAC128 mice, where no changes were found in none of the gangliosides analyzed (Figures 4D–F).

**Figure 5:**
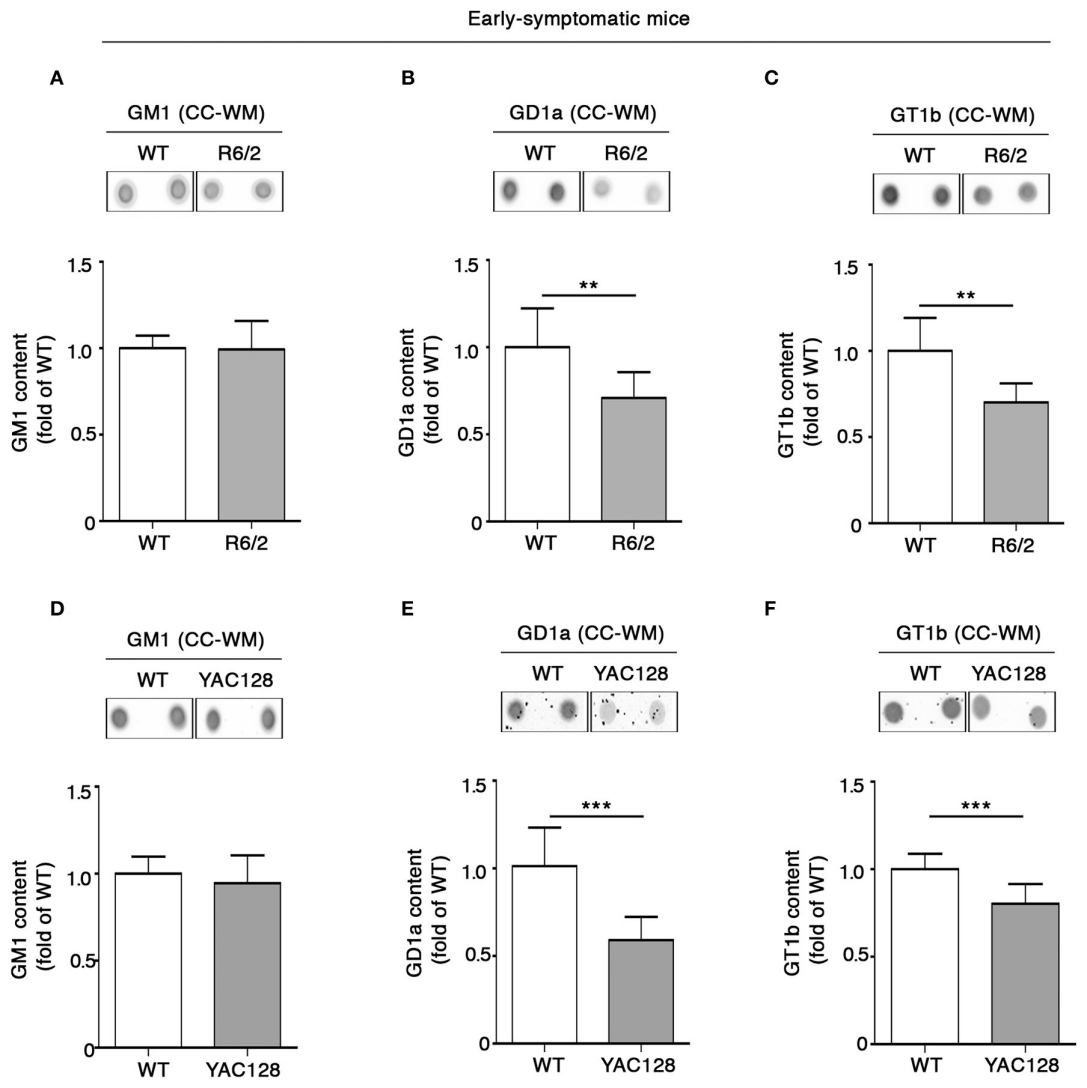
**Levels of gangliosides GD1a and GT1b are reduced in CC-WM from early symptomatic R6/2 and YAC128 HD mice**. Representative dot blotting and densitometric analysis of gangliosides GM1, GD1a, and GT1b in CC-WM from early symptomatic R6/2 (6 week old) **(A–C)** and YAC128 (5 month old) **(D–F)** and age-matched WT littermates. Ganglioside spots were visualized by ECL. Data are represented as the mean ± SD, *n* = 5 for each group of mice. ^**^P < 0.001; ^***^P < 0.0001 (non-parametric Mann–Whitney *U*-test).

Coherent with our expectation, analysis of ganglioside content in tissues from early symptomatic (6 week old) R6/2 mice confirmed the reduction of GD1a content observed at the pre-symptomatic stage (Figure 5B) and revealed that such a reduction spread also to GT1b (Figure 5C). Likewise, YAC128 mice also exhibited significant reduction of both GD1a and GT1b at similar disease stage (5 month old mice) (Figures 5E,F). No variations in GM1 content were observed either in R6/2 or in YAC128 mice (Figures 5A,D) not even at this disease stage.

## Discussion

In this study, we confirmed our previous finding of altered glycolsphyngolipid metabolism in HD and importantly highlighted, for the first time, the evidence that such a defect is not confined to the striatal and cortical tissues, but rather extended also to other brain regions like CC-WM, that has been reported being strongly implicated in the pathophysiology of HD (Rosas et al., 2010; Bohanna et al., 2011; Di Paola et al., 2012). Interestingly, the generalized reduction of GM1 in all brain tissues from both symptomatic HD mice was associated with perturbed regulation of other two specific gangliosides GD1a and GT1b, whose content was differentially distributed among striatum, cortex and CC-WM. These findings found support on previous studies reporting a brain region-changing profile for both GD1a and GT1b in both HD animals and human patients (Desplats et al., 2007; Denny et al., 2010; Maglione et al., 2010). Unlike what happens in other brain regions, CC-WM showed a concomitant and significant reduction of all three gangliosides in the symptomatic stage of the disease in both animal models. Interestingly, first signs of aberrant ganglioside metabolism in CC-WM were represented by a selective reduction of GD1a and GT1b content and were first detected in early-symptomatic stage of the disease. Moreover, GD1a content was significantly perturbed even before any disease symptoms appeared in R6/2 mice and no variation was detected in pre-symptomatic YAC128 mice. Although not clear yet, one of the possible factors that may lead to such a differential disease stage-dependent decrease of ganglioside levels, may be related to the effect on mutant Htt on the regulation of gene expression and/or activity of enzymes involved in the synthesis and degradation of each ganglioside, already reported in both HD animal models and human patients (Desplats et al., 2007; Maglione et al., 2010; Denny et al., 2010).

This hypothesis could likely explain also the slight difference between R6/2 and YAC128 mice in the timing when signs of ganglioside deficiency first appear.

Although we cannot establish a definitive correlation between ganglioside composition and the CC-WM abnormalities reported in HD (Lerch et al., 2008; Rosas et al., 2010; Bohanna et al., 2011; Di Paola et al., 2012; Di Pardo et al., 2014), we certainly speculate that the early reduction of GD1a and GT1b may conceivable contribute to callosal axon disorganization and, more in general, to the early axonal dysfunction and degeneration that may precede neuronal loss in HD pre-clinical models (Li et al., 2001; Lerch et al., 2008; Gatto et al., 2015) and eventually support the evidence of “dying-back” pattern of neurodegeneration in HD (Han et al., 2010). In the light of that, we hypothesize that the axonal dysfunction potentially resulting from ganglioside deficiency may represent the molecular event underlying the impairment of brain connectivity occurring in HD patients and in HD mutation-carriers (Rosas et al., 2010; Di Paola et al., 2012; Dumas et al., 2013; Poudel et al., 2014).

Collectively, our data confirmed the aberrant ganglioside metabolism in HD, supported the idea that gangliosides are relevant determinants in the pathogenesis of the disease and importantly, for the first time, highlighted an early, and gradual perturbation of ganglioside content in the CC-WM in two HD transgenic mouse models. However, further studies are now needed to definitely clarify any functional role of ganglioside perturbation in the onset and progression of white matter abnormalities in HD and to understand how specific CC-WM drug targets can be approached in the future.

## Author contributions

AD and EA contributed equally to this work. VM conceived and designed the study. VM and AD jointly directed the study and co-wrote the manuscripts. AD managed animal colonies and designed all the *in-vivo* experiments. EA performed all the biochemical experiments. All the authors analyzed and discussed the data and approved the manuscript.

## Funding

This research was supported by Marie Curie International Incoming Fellowship (PIIF-GA-2011-300197) granted to VM within the 7th European Community Framework Program.

### Conflict of interest statement

The authors declare that the research was conducted in the absence of any commercial or financial relationships that could be construed as a potential conflict of interest.
