# Supplementary material for: Impaired Levels of Gangliosides in the Corpus Callosum of Huntington Disease Animal Models
Source: Front Neurosci. 2016 Oct 6;10:457. doi: 10.3389/fnins.2016.00457 (PMC5052274; doi:10.3389/fnins.2016.00457)
Supplement: Supplementary file 1 [file DataSheet1.PDF]

Supplementary Material

Impaired levels of gangliosides in the Corpus Callosum of Huntington Disease animal models

Alba Di Pardo[1], Enrico Amico[1], and Vittorio Maglione*

IRCCS Neuromed, Localita' Camerelle 86077 Pozzilli (IS), Italy

[1] These authors contributed equally to this work

***Correspondence**

Vittorio Maglione

E-mail: vittorio.maglione@neuromed.it

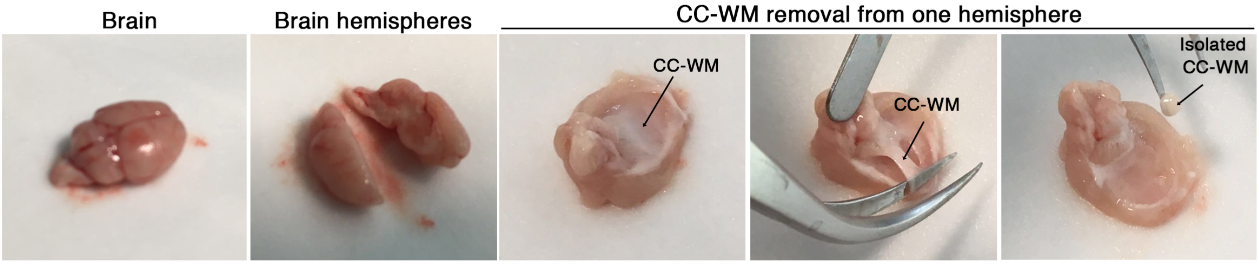

Supplementary Figure 1. Corpus Callosum (CC-WM) isolation from mouse brain.

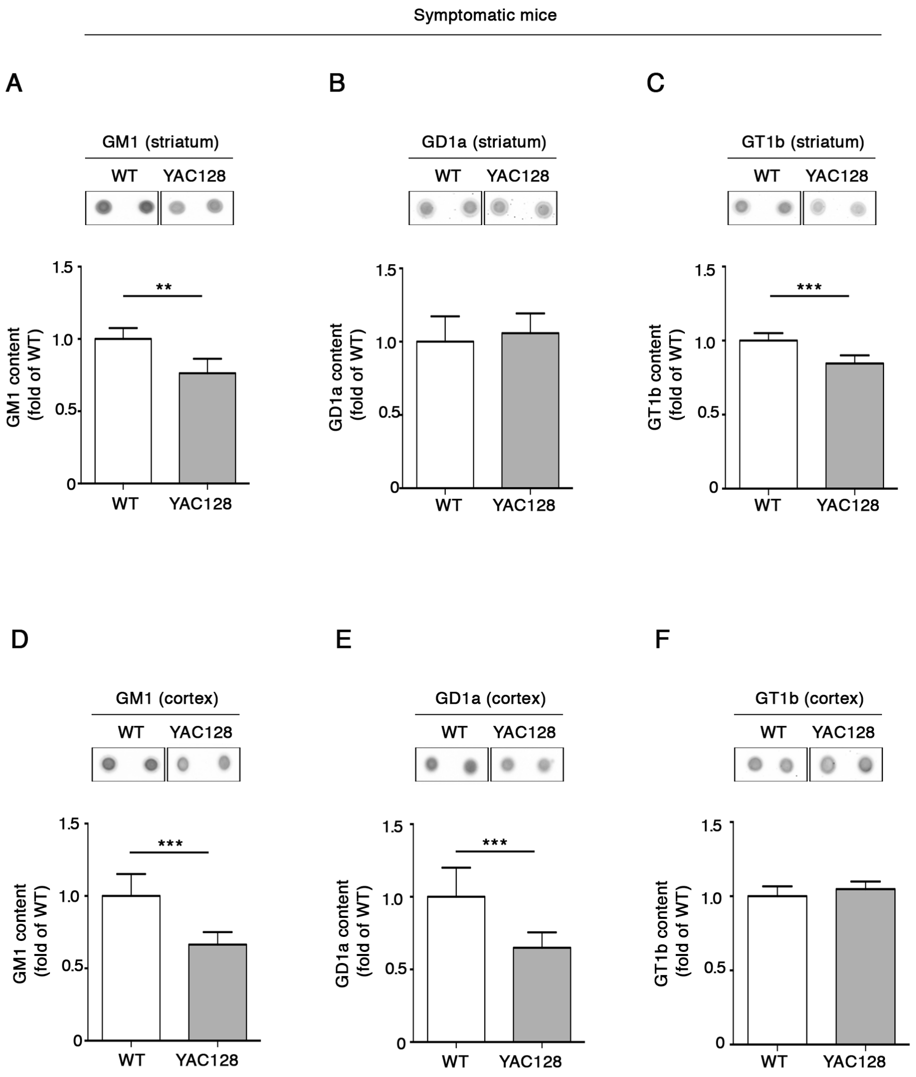

Supplementary Figure 2. Brain ganglioside content is aberrant in striatal and cortical tissues of symptomatic YAC128 HD mice. Representative dot blottings and densitometric analysis of GM1, GD1a and GT1b gangliosides in striatum (A-C) and cortex (D-E) from symptomatic (9 month old) YAC128 mice and age-matched WT littermates. Ganglioside spots were visualized by ECL. Data are represented as the mean±SD, n= 5 for each group of mice. **P<0.001; ***P<0.0001 (non-parametric Mann–Whitney U-test).

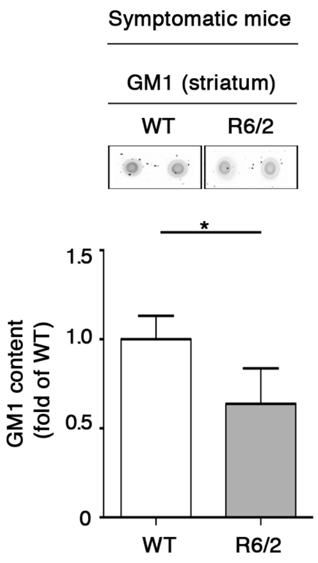

Supplementary Figure 3. Levels of ganglioside GM1 are reduced in the striatum of symptomatic R6/2 HD mice.

Representative dot blottings and densitometric analysis of GM1 in striatal tissues isolated from symptomatic R6/2 mice and WT controls. Ganglioside spots were visualized by ECL. Data are represented as the mean±SD, n= 7 for each group of mice. *P<0.05 (non-parametric Mann–Whitney U-test).
